# Head movement, an important contributor to human cerebrospinal fluid circulation

**DOI:** 10.1038/srep31787

**Published:** 2016-08-19

**Authors:** Qiang Xu, Sheng-Bo Yu, Nan Zheng, Xiao-Ying Yuan, Yan-Yan Chi, Cong Liu, Xue-Mei Wang, Xiang-Tao Lin, Hong-Jin Sui

**Affiliations:** 1 Department of Anatomy, Dalian Medical University, Dalian, 116044, China; 2 Department of Radiology, The 403 Affiliated Hospital of Chinese PLA General Hospital, Dalian, 116021, China; 3 Department of Radiology, Dalian Municipal Central Hospital, Dalian, 116033, China; 4 Shandong Medical Imaging Research Institute, School of Medicine, Shandong University, Jinan, 250021, China

## Abstract

The suboccipital muscles are connected to the upper cervical spinal dura mater via the myodural bridges (MDBs). Recently, it was suggested that they might work as a pump to provide power for cerebrospinal fluid (CSF) circulation. The purpose of this study was to investigate effects of the suboccipital muscles contractions on the CSF flow. Forty healthy adult volunteers were subjected to cine phase-contrast MR imaging. Each volunteer was scanned twice, once before and once after one-minute-head-rotation period. CSF flow waveform parameters at craniocervical junction were analyzed. The results showed that, after the head rotations, the maximum and average CSF flow rates during ventricular diastole were significantly increased, and the CSF stroke volumes during diastole and during entire cardiac cycle were significantly increased. This suggested that the CSF flow was significantly promoted by head movements. Among the muscles related with head movements, only three suboccipital muscles are connected to the upper cervical spinal dura mater via MDBs. It was believed that MDBs might transform powers of the muscles to CSF. The present results suggested that the head movements served as an important contributor to CSF dynamics and the MDBs might be involved in this mechanism.

The suboccipital area consists of complex anatomical structures. The myodural bridge (MDB) as dense soft tissue communication extends from the suboccipital musculatures to the cervical spinal dura mater^1^,^2^,^3^,^4^,^5^,^6^,^7^,^8^,^9^,^10^,^11^,^12^,^13^,^14^,^15^. Among the suboccipital muscles, the rectus capitis posterior minor (RCPmi)^1^,^2^,^3^,^4^,^5^,^6^, the rectus capitis posterior major (RCPma), the obliquus capitis inferior (OCI)^7^,^8^,^9^,^10^,^11^ give off the MDB respectively. The functional significance of the MDB is still a matter of research and debate. Certain studies proposed that MDBs might generally confer a mechanical advantage to the suboccipital muscles by protecting the spinal cord from dural enfolding^1^,^3^,^4^,^5^,^7^,^8^,^10^. It was also suggested that the suboccipital muscle’s reflexive myostatic response could be involved in placing the dura under tension via the MDB^7^,^8^,^10^,^11^ and that the myodural biofeedback might participate in maintaining the integrity of the subarachnoid space^7^,^8^. Based on our recent study, a novel hypothesis on the physiological function of MDBs was proposed that suboccipital muscles might work as a pump via the MDBs to provide power for CSF circulation^14^,^15^. In order to provide an evidence supporting this pump hypothesis, CSF flow at craniocervical junction was investigated by using cine phase-contrast (cine-PC) MR imaging before and after one-minute-head-rotation period in this study.

## Results

**Figure 1:**
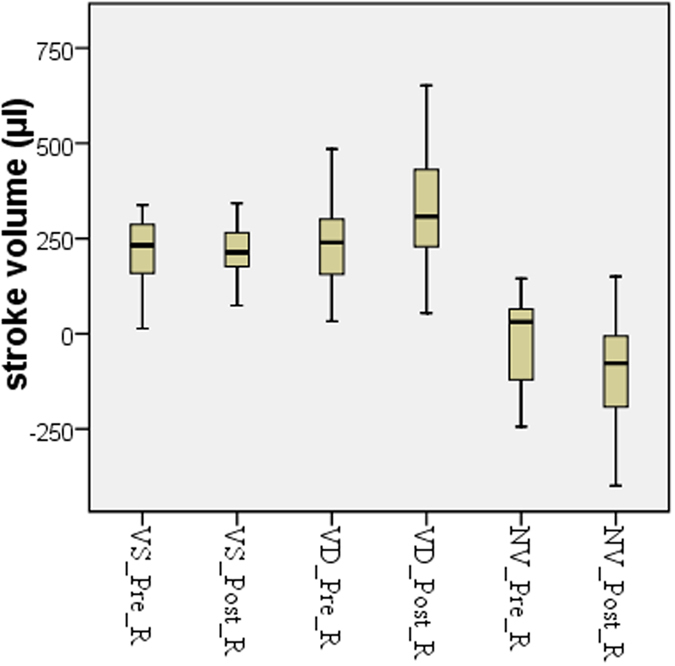
The CSF dynamics volumetric parameters were showed in Box and Whisker plots. Outliers were removed from forty cases. VS_Pre_R (Pre-rotation VS) and VS_Post_R (Post-rotation VS), n = 38; VD_Pre_R (Pre-rotation VD) and VD_Post_R (Post-rotation VD), n = 37, *p* = 0.001; NV_Pre_R (Pre-rotation NV) and NV_Post_R (Post-rotation NV), n = 39, *p* = 0.003.

The cardiac cycle-related CSF flow pulsations were depicted as CSF flow waveforms. Based on the waveforms, the CSF flow quantitative parameters of the craniocervical junction were calculated. And the results of both scans and the comparison between them are presented in Tables 1, 2, 3, 4 and Fig. 1.

### Temporal analysis

**Figure 2:**
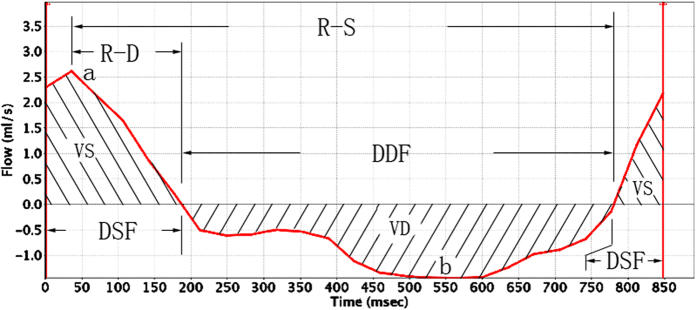
Example of a CSF flow rate waveform from one of the volunteers, with a graphical representation for most parameters analyzed. Positive waveform values correspond to systolic (craniocaudal orientation) CSF flow, whereas negative values correspond to diastolic (caudocranial orientation) CSF flow. R-D, the interval ranging from the R wave to the onset of the diastolic CSF flow; R-S, the interval ranging from the R wave to the onset of the systolic CSF flow; DSF, duration of the CSF systolic flow; DDF, duration of the CSF diastolic flow. a, peak of the systolic curve, representing the maximum systolic flow rate (MSFR); b, trough of the diastolic curve, representing the maximum diastolic flow rate (MDFR); VS, area under the systolic curve, representing the CSF flow volume during the systole; VD, area under the diastolic curve, representing the CSF flow volume during the diastole. In addition, some parameters obtained by calculations, the average systolic flow rate (ASFR) = VS/DSF, the average diastolic flow rate (ADFR) = VD/DDF, and the CSF stroke volume during the entire cardiac cycle (net flow volume, NV) = VS + VD.

The temporal CSF dynamics parameters were presented in the Table 1 and included the mean values measured for the interval ranging from the R wave (peak of positive curve) to the onset of the diastolic CSF flow (R-D), the interval ranging from the R wave to the onset of the systolic CSF (R-S), the duration of the CSF systolic flow (DSF) and the duration of the CSF diastolic flow (DDF) (Fig. 2). No statistically significant difference in the temporal parameters was observed between the pre- and post-head rotation measurements (Table 1).

### Amplitude analysis

The CSF dynamics amplitude parameters were presented in the Table 2 and included the mean values of the maximum systolic flow rate (MSFR), the maximum diastolic flow rate (MDFR), the average systolic flow rate (ASFR) and the average diastolic flow rate (ADFR) (Fig. 2). The MDFR and the ADFR were significantly increased after the one-minute-head-rotation period, but the MSFR and the ASFR remained unchanged (Table 2).

### Volumetric analysis

The CSF dynamics volumetric parameters included the mean values of the systolic CSF flow volume (VS), the diastolic CSF flow volume (VD) and the net-flow volume (CSF stroke volume during the entire cardiac cycle, NV) (Fig. 2) and were presented in the Table 3. The value distribution of these parameters was showed in Fig. 1. The VD was significantly increased after the one-minute head rotation period, but the VS remained unchanged. As a result of the increase in the VD values, the NV was significantly increased in cranial direction after the one-minute head rotation period.

Furthermore, the detailed CSF stroke volume variations after the one-minute-head-rotation period were analyzed and results were presented in Table 4. Before the head rotation period, the orientation of the NV value represented a caudal direction in 22 subjects and a cranial direction in 18 subjects out of the 40 volunteers. In the subgroup with an initial caudal NV direction, the NV values were strengthened in the cranial orientation following the one-minute head rotation period in twenty subjects (20/22), whereas the NV values were strengthened in the caudal orientation in only two subjects (2/22). On the other, in the subgroup with an initial cranial NV direction, the NV values were strengthened in the cranial orientation in seven subjects (7/18) after the one-minute head rotation period, whereas the NV values were strengthened in the caudal orientation in eleven subjects (11/18). The change in the NV after the one-minute-head-rotation period differed significantly between both subgroups (*p* value, 0.002).

In the above results, among forty cases, a small number of measurements were found as outliers based on Box and Whisker Plots analysis. It was impossible to re-measure the subjects with outliers, so these outliers were not included in T-test. Additionally, the intervals between the one-minute-head-rotation and the second cine-PC scan ranged from 1.0–3.6 minutes, with a mean value of 1.9 ± 0.57 minutes, because of the process of re-positioning MRI scan ahead of the second cine-PC scan. During the scanning procedure, the heart beat of each subject was monitored. The mean heart rate values before and after the head rotation period were of 70.7 ± 8.8 and 69.9 ± 7.3 beats per minute, respectively, showing no significant change in heart rate after the head rotation period (*p* value 0.320).

## Discussion

The MDBs are dense connections extending from the suboccipital structures to the cervical dura sac^1^,^2^,^3^,^4^,^5^,^6^,^7^,^8^,^9^,^10^,^11^,^12^,^13^,^14^,^15^. The functional significance of the MDB is still a matter of research and debate^1^,^3^,^4^,^5^,^7^,^8^,^10^,^11^,^14^,^15^. Recently a novel hypothesis on the physiological function of MDBs was proposed that suboccipital muscles might work as a pump via the MDBs to provide power for CSF circulation^14^,^15^.

This study assessed various parameters of the CSF flow waveform and provided an analysis of CSF flow pulsations in the entire cross-sectional area of the subarachnoid space at the level of the atlas upper border. Herein, CSF flow parameters were compared before and after one-minute-head-rotation period in 40 healthy volunteers. The results demonstrate that CSF diastolic flow is significantly affected by the one-minute-head-rotation period. After the head rotation period, the MDFR was significantly increased from 0.81 ± 0.34 to 0.98 ± 0.36 ml/s, meanwhile the ADFR was significantly increased from 0.43 ± 0.20 to 0.56 ± 0.24 ml/s (Table 2). As a result of the increased diastolic CSF flow rate, the VD was found significantly increased after the head rotation period, from 232.5 ± 112.7 to 323.6 ± 143.9 μl, and the NV was found significantly increased from −19.5 ± 118.4 to −113.2 ± 150.1 μl after the head rotation period (Table 3).

Furthermore, the effects of head rotation on CSF circulation were different according to the initial NV flow direction before the head rotation period. In 90.9% of the subjects with an initial caudal NV flow direction, the NV flows were strengthened in the cranial orientation following the one-minute head-rotation period. In 61.1% of the subjects with an initial cranial NV flow direction, however, the NV flows were strengthened in the caudal orientation (Table 4). It meant that the effects of head rotations on the CSF circulation had individual differences, which depended on initial conditions of the CSF circulation before the head rotations.

In addition, above experimental results were detected 1.0–3.6 minutes after the one-minute head-rotation period because of the process of re-positioning MRI scan. These results implied that head rotations had markedly carry-over effect on the CSF circulation probably based on some mechanisms, e.g. motion inertia. The immediate effect of the head rotation might be more remarkable than its carry-over one. In future, it might be detected by recently developed real-time MRI techniques^16^.

It is well known that the total CSF volume in the adult is approximately 130 ml, of which about 60 ml is contained within the cranial cavity, and about 70 ml is stored within the vertebral canal^17^. A large amount of CSF runs through the craniocervical junction. In present study, we found that the CSF dynamics at craniocervical junction were significantly changed by the one-minute-head-rotation. Especially, the stroke volume during the entire cardiac cycle was increased significantly in cranial direction after the head rotations. Thus it was speculated that head movements could be a significant contributor to CSF dynamics in the craniocervical junction, besides mentioned factors in the past, such as heartbeat^18^,^19^ and respiration^20^,^21^,^22^.

There are many muscles in the neck related with head movements. Among these muscles, the RCPmi, RCPma and OCI are connected to the upper cervical spinal dura mater via the MDBs and vertebral dural ligament^1^,^2^,^3^,^4^,^5^,^6^,^7^,^8^,^9^,^10^,^11^,^12^,^13^,^14^,^15^. During the head rotation, the sub occipital muscles could draw the cervical dural sleeve by the MDB and then head movements might work like a muscle pump which drives CSF based on deformations of the dural sac. The results of this study provided a strong evidence to support the MDB functional hypothesis of Sui^14^ and Zheng^15^.

Except for the MDB connecting the head movements and the cervical dura mater, many other factors, for example head movement probably induced changes of the heart beat and respiration, etc., would likely be involved. It was well known that the heart beat is an important factor effecting CSF circulation^18^,^19^. In the present study the CSF dynamics temporal parameters throughout the cardiac cycle and the amplitude parameters (MSFR, ASFR and VS) during the ventricular systole were found to remain unchanged after the one-minute-head-rotation period (Tables 1, 2, 3). Combined that the volunteers’ heart beats were 70.7 ± 8.8 and 69.9 ± 7.3 bpm before and after the head rotation period respectively with no significant change between them, it was concluded that the effects of the heart beat on the CSF circulation were maintained constantly after the one-minute head-rotation period. As well as heart beats, respiration was found to affect the CSF flow in humans^20^,^21^,^22^. In present study, each volunteer was required to perform continuous head rotations for a period of 60 seconds, while lying on their back on the MRI bed and their heads against the bed. For this was a light activity and meanwhile the effects of heart on the CSF circulation were maintained constantly after the one-minute head-rotation period, the effect of changes of respiration on the CSF circulation was speculated to be small.

Regarding the current prevailing clinical opinions, the hyperkinetic CSF flow in the Chiari malformation might result in associated signs and symptoms in patients, including syringomyelia, headaches, and other neurological findings^23^,^24^. And the clinical relevance of the relationships between myodural bridges and cervicogenic or tension headaches syndromes was also proposed previously^25^,^26^,^27^,^28^,^29^,^30^,^31^,^32^. In this regard our results might provide a new explanation for the pathophysiology of cervicogenic headaches based on the relationship of CSF circulation and head movements. Our recent study found that chronic headaches were correlated with the RCPmi. Patients with chronic headaches suffered from more obvious hypertrophy than that of the control group^33^. It might be evidence supporting our assumption in the clinical manner.

In conclusion, the present investigation demonstrated that CSF circulation in the craniocervical junction is significantly propelled in cranial orientation by head rotations, and head movement is a novel important contributor to CSF dynamics. The connections between the upper cervical spine dura mater and the suboccipital musculatures, especially the MDBs, might be involved in above mechanism.

## Methods

### Materials

Approval from the Ethics Committee for Research at the Basic Medical College of Dalian Medical University was obtained for this study. The experimental protocol of this study was carried out in accordance with the approved guidelines. In this study, a group of forty adult volunteers (16 men, 24 women; aged 20–49 years; mean age, 26.2 ± 7.2 years), including most researchers involved in this study, was subjected to MR imaging (1.5T scanner, GE). Informed consent was obtained from all of them and none of them had any history of cardiovascular, neurological, endocrine and cervical disorders.

### Cine-PC MR imaging

A cine-PC MR imaging method^16^ was used in this study to measure the cardiac-gated CSF flow through the transverse plane, at the level of the atlas upper border with a peripheral pulse trigger (e.g., finger photoplethysmography). Imaging parameters were collected as follows: TR, 33 ms; TE, 10 ms; flip angle, 20°; imaging matrix, 256 × 192; FOV, 240 cm^2^; section thickness, 5 mm; and 2 signal intensity average. The encoding direction was in the head-to-foot orientation for all volunteers. PC- images were obtained at each time points, for a total of 25 measurements equally distributed over the cardiac cycle. The imaging time duration varied between 2 to 4 minutes, depending on the volunteer’s heart rate.

**Figure 3:**
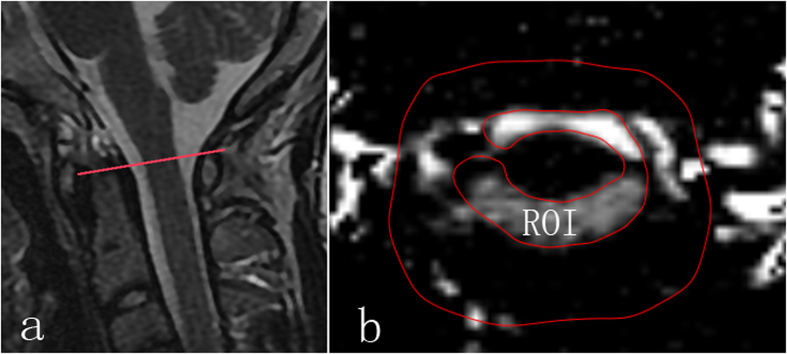
Example of MR images from one of the volunteers. The region of interest was the entire cross-sectional area of the subarachnoid space at the level of the atlas upper border. (**a**) Showing the level of upper border of the atlas (red line) in the median sagittal plane of head and neck; (**b**) the region of interesting (ROI, inner closed irregular contour) showing the entire cross-sectional area of the subarachnoid space in the transverse plane at the level of the atlas upper border. And the outer red line served as a guide line.

Before the cine-PC scan, sagittal T2 weighted images of head and neck were obtained by a quick MR scan to provide anatomic details. Based on the median sagittal images, a transverse plane for the cine-PC scan was designated at the level of the atlas upper border (Fig. 3a).

Each volunteer was scanned twice. The first scanning was performed after a resting period. After that, each volunteer was required to perform continuous head rotations for a period of 60 seconds, while lying on their back on the MRI bed and their heads against the bed. The head rotation speed was set at around 0.5 cycles per second. One head rotation cycle was counted when the head was rotated from median position to one side and next by another side and then return. Volunteers turned their heads according to the researcher’s instructions for normalization of the one-minute-head-rotation. A scan for re-positioning underwent immediately after the one-minute-head-rotation period and then the second cine-PC scan followed.

Cine-PC images were transferred to an independent workstation (aw46mr) for CSF flow analysis using the Analyze (Report Card 4.0) software. Using the region-of-interest function of the Analyze software, an irregular contour was drawn manually to encompass the entire cross-sectional area of the subarachnoid space at the level of the atlas upper border (Fig. 3,b). The region-of-interest statistics output from the Analyze software included the mean velocity values (ml/s) [(the average speed of all pixels in the ROI) * (the area of the ROI)] at each time point during the cardiac cycle. Results were plotted as waveforms with flow rate on the y-axis and cardiac cycle fractions on the x-axis (Fig. 2). On the waveform, positive values corresponded to systolic (craniocaudal orientation) CSF flow and negative values corresponded to diastolic (caudocranial orientation) CSF flow. The CSF flow rate waveforms were analyzed according to the temporal and amplitude parameters (Fig. 2).

### Data Analysis

The range, mean, and standard deviation were calculated for each parameter. All parameters were compared between both scans acquired before and after the one-minute head rotation period. Statistical significance was calculated using a paired sample t-test or Chi-Square test. A *p* value of less than 0.05 indicated a statistically significant difference.

## Additional Information

**How to cite this article**: Xu, Q. *et al.* Head movement, an important contributor to human cerebrospinal fluid circulation. *Sci. Rep.* **6**, 31787; doi: 10.1038/srep31787 (2016).

## Figures and Tables

**Table 1:** Comparison of the temporal parameters obtained before and after the head rotation period.

Values	R-S		R-D		DSF		DDF	
	Pre-rotation	Post-rotation	Pre-rotation	Post-rotation	Pre-rotation	Post-rotation	Pre-rotation	Post-rotation
N	35		38		38		40	
Range	509.0~972.9	546.6~961.5	60.7~444.6	39.7~375.8	157.8~574.7	174.4~417.5	231.9~781.1	331.2~776.8
Mean ± SD	714.9 ± 110.9	731.5 ± 101.2	234.5 ± 94.6	208.4 ± 73.9	317.3 ± 97.53	288.4 ± 66.84	503.8 ± 107.37	539.5 ± 107.88
t Value	−0.985		1.553		1.745		−1.751	
*p* Value	0.332		0.129		0.089		0.088	

Note: Temporal values were given in milliseconds. The outliers were removed on the base of Box and Whisker Plots analysis.

**Table 2:** Comparison of the amplitude parameters obtained before and after the head rotation period.

Values	MSFR		MDFR		ASFR		ADFR	
	Pre-rotation	Post-rotation	Pre-rotation	Post-rotation	Pre-rotation	Post-rotation	Pre-rotation	Post-rotation
N	39		36		39		34	
Range	0.94~3.40	0.68~3.65	0.27~1.47	0.26~1.75	0.05~1.64	0.11~1.32	0.07~0.89	0.16~1.07
Mean ± SD	2.11 ± 0.60	2.11 ± 0.63	0.81 ± 0.34	0.98 ± 0.36*	0.75 ± 0.33	0.75 ± 0.30	0.43 ± 0.20	0.56 ± 0.24*
t Value	−0.048		−2.400		0.093		−2.654	
*p* Value	0.962		0.022		0.926		0.012	

Note: Amplitude values were given in milliliter per second. The outliers were removed on the base of Box and Whisker Plots analysis. Stars show significantly difference.

**Table 3:** Comparison of the volumetric parameters acquired before and after the head rotation period.

Values	VS		VD		NV	
	Pre-rotation	Post-rotation	Pre-rotation	Post-rotation	Pre-rotation	Post-rotation
N	38		37		39	
Range	13.4~365.7	73.9~342.8	33.0~−485.0	54.2~−651.0	−356.9~145.1	−519.2~150.2
Mean ± SD	227.5 ± 81.8	218.7 ± 63.7	232.5 ± 112.7	323.6 ± 143.9*	−19.5 ± 118.4	−113.2 ± 150.1*
t Value	0.558		−3.495		3.227	
*p* Value	0.580		0.001		0.003	

Note: Volumetric values are given in microliter. The negative value above means flowing in cranial direction. The outliers were removed on the base of Box and Whisker Plots analysis. Stars show significantly difference.

**Table 4:** Change tendency of CSF stroke volume following the one-minute-head-rotation period in two initial orientation subgroups (n = 40).

Pre-rotation	Post-rotation		Total
	Strengthened in cranial orientation	Strengthened in caudal orientation	
Caudal direction	20 (90.9%)	2 (9.1%)	22 (100%)
Cranial direction	7 (38.9%)	11 (61.1%)	18 (100%)
Total	27	13	40

Note: Chi-Square Test, χ^2^ = 9.956 (continuity correction), *p* value = 0.002.
